# ^18^F-FDG PET/CT assessment of metabolic tumor burden predicts survival in patients with metastatic posterior uveal melanoma

**DOI:** 10.1038/s41598-025-88625-w

**Published:** 2025-02-03

**Authors:** Tine Gadegaard Hindso, Torben Martinussen, Camilla Wium Bjerrum, Sune Høgild Keller, Annika Loft, Mette Bagger Sjøl, Kristoffer Nissen, Carsten Faber, Marco Donia, Inge Marie Svane, Eva Ellebaek, Steffen Heegaard, Jens Folke Kiilgaard, Karine Madsen

**Affiliations:** 1 Department of Ophthalmology, Copenhagen University Hospital – Rigshospitalet, Blegdamsvej 9, Copenhagen Ø, 2100 Denmark; 2 Department of Biostatistics, University of Copenhagen, Øster Farimagsgade 5, Copenhagen K, 1014 Denmark; 3 Department of Radiology, Copenhagen University Hospital – Rigshospitalet, Blegdamsvej 9, Copenhagen Ø, 2100 Denmark; 4 Department of Clinical Physiology and Nuclear Medicine, Copenhagen University Hospital – Rigshospitalet, Blegdamsvej 9, Copenhagen Ø, 2100 Denmark; 5 Department of Oncology, National Center for Cancer Immune Therapy (CCIT-DK), Copenhagen University Hospital – Herlev and Gentofte, Borgmester Ib Juuls Vej 13, Herlev, 2730 Denmark; 6 Department of Pathology, Copenhagen University Hospital – Rigshospitalet, Blegdamsvej 9, Copenhagen Ø, 2100 Denmark

**Keywords:** Uveal melanoma, FDG PET, Metabolic tumor burden, Metabolic tumor volume (MTV), Total lesion glycolysis (TLG), Survival, Biomarkers, Oncology, Prognostic markers

## Abstract

The prognostic value of metabolic tumor burden parameters obtained from ^18^F-FDG PET/CT imaging was evaluated in this retrospective national multicenter study of patients with metastatic posterior uveal melanoma (PUM) and compared to the largest diameter of the largest metastatic lesion (LDLM) and the American Joint Committee on Cancer (AJCC) staging system. The Maximal Standard Uptake Value (SUV_max_), Metabolic Tumor Volume (MTV), and Total Lesion Glycolysis (TLG) were obtained in 106 patients. Higher values of SUV_max_ (*p* = 0.007, log-rank), MTV (*p* < 0.001, log-rank), and TLG (*p* < 0.001, long-rank) were associated with shorter survival. The three parameters were also independent predictors in the multivariate Cox model, while the AJCC staging turned insignificant. Time-dependent positive predictive value (PPV) analysis and Receiver Operating Characteristics (ROC) curves showed that MTV (Area Under the Curve (AUC) = 0.78), TLG (AUC = 0.78), and LDLM (AUC = 0.76) were good predictors of 1-year survival. For the subset of 97 patients with liver metastases, the corresponding regional measurements in the liver tended to be even better predictors. In conclusion, MTV and TLG were found to be better predictors of survival in metastatic PUM than the AJCC staging system, but when LDLM was used as a continuous variable it showed an equally good prediction of 1-year survival.

**Supplementary Information:**

The online version contains supplementary material available at 10.1038/s41598-025-88625-w.

## Introduction

Posterior uveal melanoma (PUM) can arise in the choroid or the ciliary body. It affects about 1–11 cases per million person-years, with the highest incidence rates in Australia, New Zealand, and the Northern European countries^1,2^. About half of the patients develop metastatic disease^3^, and once diagnosed with metastases from PUM, patients have a median survival of only 12 months^4^. For reasons yet to be explored, PUM has a high tendency to disseminate to the liver, and hepatic metastases are found in about 90% of the patients with distant metastases^5,6^. In metastatic PUM, [^18^F]-fluorodeoxyglucose positron emission computed tomography (^18^F-FDG PET/CT) plays a well-established role in staging and detection of extrahepatic lesions, and it is used to guide treatment decisions and monitor treatment effects^7–11^. In the screening for metastatic development, liver ultrasonography and magnetic resonance imaging (MRI) have proved to be superior to computed tomography (CT)^12^, and due to radiation exposure and the low frequency of exclusive extrahepatic lesions, ^18^F-FDG PET/CT is not utilized for screening in Denmark. ^18^F-FDG PET/CT imaging has little value in the primary staging of PUM, as only 2.8–3.8% of patients have metastatic disease at the time of primary diagnosis^7,11^.

In the clinical setting, ^18^F-FDG PET/CT imaging provides important information about the size, localization, and intensity of the metabolic activity of malignant lesions^13^, but quantitative measurements of the metabolic tumor burden can also be assessed: the maximal Standardized Uptake Value (SUV_max_, highest concentration of ^18^F-FDG uptake within the tumor tissue), the Metabolic Tumor Volume (MTV, the summed volume of the metabolically active tumor tissue accumulating ^18^F-FDG), and the Total Lesion Glycolysis (TLG, defined as MTV multiplied by the mean ^18^F-FDG uptake within the tumor tissue (SUV_mean_))^14–16^. As TLG includes estimates of both the tumor volume and the metabolic activity, TLG is often considered the optimal parameter of tumor burden^17,18^. TLG, MTV, and, to a lesser extent, SUV_max_, have been identified as prognostic biomarkers in various primary cancers^17,19,20^, and in the metastatic setting^18,21–23^. In metastatic uveal melanoma, the prognostic role of SUV_max_ has been investigated by del Carpio et al.^22^, who found that increasing SUV_max_ in metastatic liver lesions was associated with poorer survival outcomes. Eldredge-Hindy et al. found that pretreatment MTV and TLG, but not SUV_max_, of only intrahepatic metastatic PUM tumors were associated with survival, whereas SUV_max_ was not associated with survival outcomes^23^.

Today, the largest diameter of the largest metastatic lesion (LDLM) is used as a pseudo biomarker for the tumor burden, where the American Joint Committee on Cancer (AJCC) staging system stages patients into three prognostic categories according to LDLM: ≤ 30 mm (M1a), 31–80 mm (M1b), ≥ 81 mm (M1c)^24^. The LDLM is easy to obtain, but has some shortcomings, as only the size of the largest metastatic lesion is considered which might not always be correlated with the total tumor burden.

In the present study, we investigated whether whole-body SUV_max_, MTV, and TLG were stronger predictors of survival than the currently used AJCC staging system and LDLM as a continuous variable. As the majority of patients develop hepatic metastases, we further investigated whether the SUV_max_, MTV, and TLG of only the liver metastases (liver-SUV_max_, liver-MTV, and liver-TLG) were as strong or better predictors of survival than SUV_max_, MTV, and TLG of the whole body.

## Results

One hundred and six patients with metastatic PUM and an available ^18^F-FDG PET/CT were included (Table 1, Supplementary Fig. S1).

**Table 1:** Patient characteristics in 106 patients with newly diagnosed metastatic posterior uveal melanoma.

Sex, *n*	
Women	59 (56%)
Men	47 (44%)
Age at metastatic diagnosis	
Median	65 (IQR 59–73)
< 60 years of age, n	28 (26%)
≥ 60 years of age, n	78 (74%)
Time from primary diagnosis to first metastasis (months), median (IQR)	27.0 (12.0–44.0)
Time from date of metastatic diagnosis until PET/CT (days), median (IQR)	9.5 (6.0–15.0)
Overall survival (months), median (IQR)	11.5 (7.0–20.0)
^18^F-FDG PET parameter^a^, median	
SUV_max_	10.1 (IQR 6.5–15.4)
MTV (cm^3^)	14.6 (IQR 3.0-88.6)
TLG	85.5 (IQR 16.5-570.5)
Liver-SUV_max_	8.9 (IQR 5.8–13.2)
Liver-MTV (cm^3^)	13.8 (IQR 1.19–62.5)
Liver-TLG	73.6 (IQR 5.5-387.8)
LDLM (mm), median (IQR)^b^	30 (19.0–52.0)
AJCC stage IV, n	
M1a	54 (51%)
M1b	40 (38%)
M1c	12 (11%)
Metastatic pattern, n	
Hepatic pattern	62 (58%)
Hepatic-extrahepatic pattern	35 (33%)
Extrahepatic pattern	9 (8%)
WHO/ECOG performance status, n	
0–1	84 (79%)
2–4	9 (9%)
Missing values	13 (12%)
First-line ipi + nivo treatment^c^, n	
No	73 (69%)
Yes	33 (31%)
Resection of metastatic lesion(s), n	
No	90 (85%)
Yes	16 (15%)

^18^F-FDG = ^18^F-fludeoxyglucose; AJCC = American Joint Committee on Cancer; AP = alkaline phosphatase; ECOG = Eastern Cooperative Oncology Group; ipi + nivo = ipilimumab and nivolumab; IQR = interquartile range; LDH = lactase dehydrogenase; LDLM = largest diameter of the largest metastatic lesions; MBq = megaBecquerel; MTV = metabolic tumor volume; SUV = Maximum Standardized Uptake Value; TLG = tumor lesion glycolysis; UNL = upper normal limit; WHO = World Health Organization.

^a^SUV threshold = 4.0.

^b^Largest diameter of the largest metastatic lesion in any organ.

^c^First-line treatment with the combination of ipilimumab and nivolumab.

### Survival analysis

**Fig. 1:**
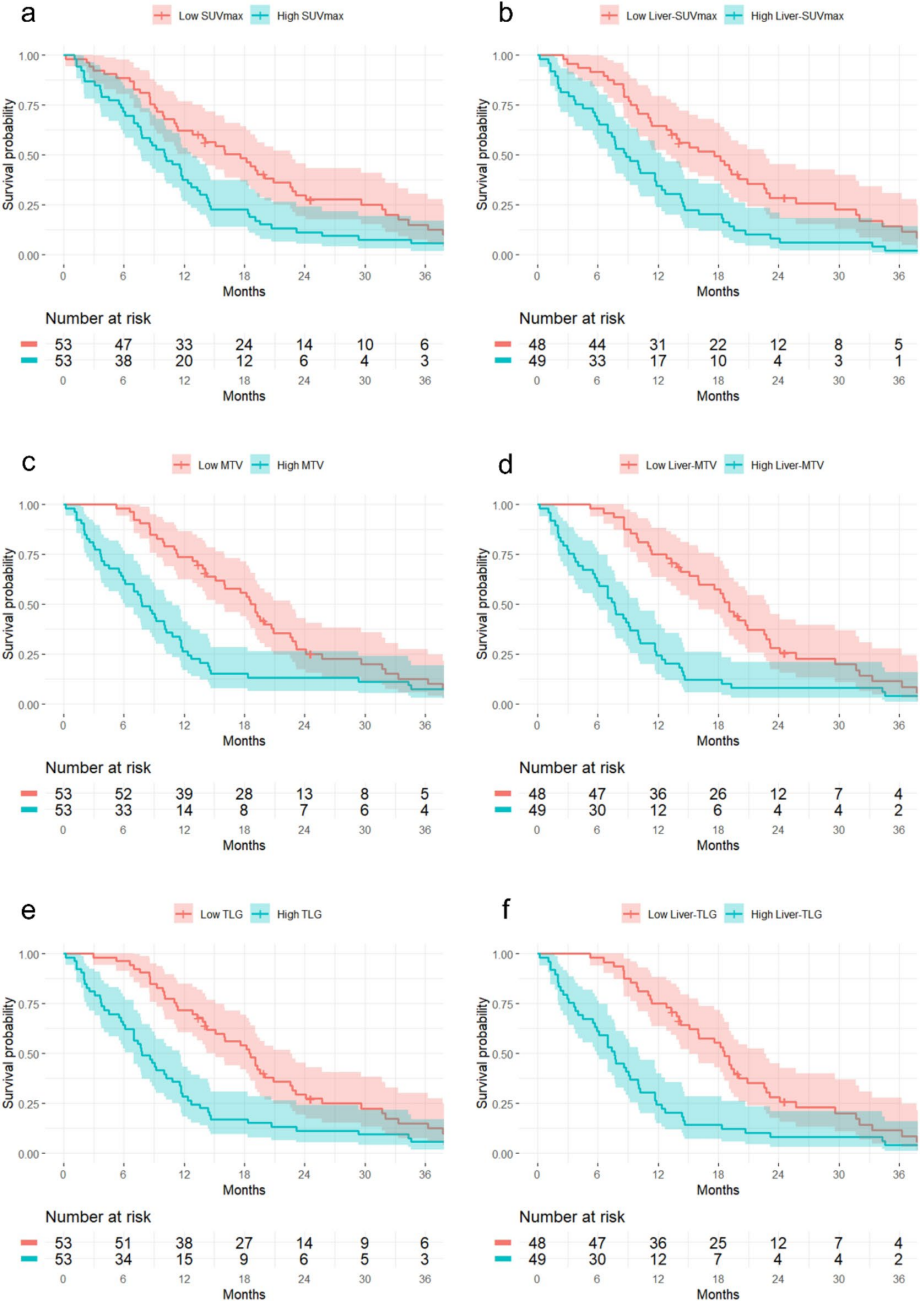
Overall survival from the date of baseline ^18^F-FDG PET/CT scan to the date of death or last follow-up in 106 patients with metastatic posterior uveal melanoma, with the x-axis limited to 36 months. **(a)** Stratified by whole-body SUV_max_ below or above the median of 10.1 (*p* = 0.007, log-rank), **(b)** stratified by liver-SUV_max_ below or above the median of 8.9 (*p* = 0.001, log-rank), **(c)** stratified by whole-body MTV below or above the median of 14.6 cm^3^ (*p* < 0.001, log-rank), **(d)** stratified by liver-MTV below or above the median of 13.8 cm^2^ (*p* < 0.001, log-rank), **(e)** stratified by whole-body TLG below or above the median of 85.5 (*p* < 0.001, log-rank), and **(f)** stratified by liver-TLG below or above the median of 73.6 (*p* < 0.001, log-rank).

The median overall survival (mOS) for the entire cohort was 11.5 months. Patients were dichotomized into high and low groups using the median values for each metabolic tumor burden parameter: The mOS was 10.0 months (95% CI: 7.0–12.0 months) in patients with SUV_max_ > the median of 10.1 compared to 17.0 months (95% CI: 12.0–22.0 months) in patients with SUV_max_ < 10.1 (Fig. 1a, *p* = 0.007, log-rank test). In patients with MTV > 14.6 cm^3^, the mOS was 7.0 months (95% CI: 6.0–10.0 months) compared to 19.0 months (95% CI: 15.0–22.0 months) in patients with MTV < 14.6 cm^3^ (Fig. 1c, *p* < 0.001, log-rank test), and in patients with a TLG > 85.5 the mOS was 7.0 months (95% CI: 6.0–11.0 months) compared to 18.0 months (95% CI: 15.0–22.0 months) in patients with TLG < 85.5 (Fig. 1e, *p* < 0.001, log-rank test).

Survival analysis was also performed on the subset of 97 patients with liver metastases, including liver-SUV_max_, liver-MTV, and liver-TLG in the analyses. In patients with liver-SUV_max_ > the median of 8.9, the mOS was 8 months (95% CI: 7–11 months) compared to 17 months (95% CI: 13–22 months) in patients with liver-SUV_max_ < 8.9 (Fig. 1b, *p* = 0.001, log-rank test). The mOS was 7 months (95% CI: 5–10 months) in patients with liver-MTV > 13.8 cm^3^ compared to 19 months (95% CI: 15–22 months) in patients with liver-MTV < 13.8 cm^3^ (Fig. 1d, *p* < 0.001, log-rank test), and the mOS was 7 months (95% CI: 5–10) in patients with liver-TLG > 73.6 compared to 18 months (95% CI: 15–22) in patients with liver-TLG < 73.6 (Fig. 1f, *p* < 0.001, log-rank test).

The univariate Cox regression analysis showed increased hazard ratio (HR) for log10-SUV_max_, sqrt-MTV, sqrt-TLG, and for the dichotomized variables (Table 2).

**Table 2:** Univariate and multivariate Cox proportional hazard regression models for overall survival from date of baseline PET-CT to death or last follow-up in 106 patients with metastatic posterior uveal melanoma.

	Univariate Cox model		Multivariate Cox model with whole-body SUVmax		Multivariate Cox model with whole-body MTV		Multivariate Cox model with whole-body TLG	
	HR (95% CI)	*p*	HR (95% CI)	*p*	HR (95% CI)	*p*	HR (95% CI)	*p*
Sex								
Women	1		1		1		1	
Men	1.10 (0.74–1.64)	0.639	1.17 (0.72–1.91)	0.519	1.13 (0.70–1.85)	0.616	1.18 (0.72–1.91)	0.508
Age								
Under 60 years of age	1		1		1		1	
Over 60 years of age	1.54 (0.98–2.44)	0.063	1.47 (0.85–2.52)	0.167	1.46 (0.83–2.55)	0.187	1.50 (0.86–2.64)	0.157
AJCC stage IV								
M1a	1		1		1		1	
M1b	1.31 (0.85–2.01)	0.216	1.76 (1.04–2.99)	**0.035**	0.98 (0.54–1.79)	0.950	0.99 (0.55–1.80)	0.976
M1c	3.22 (1.68–6.18)	**< 0.001**	5.84 (1.92–17.75)	**0.002**	0.86 (0.19–3.98)	0.848	0.81 (0.18–3.63)	0.784
Metastatic pattern								
Extrahepatic	1		1		1		1	
Hepatic	1.73 (0.80–3.72)	0.156	6.50 (2.33–18.13)	**< 0.001**	4.19 (1.64–10.73)	**0.003**	4.63 (1.80-11.88)	**0.001**
Hepatic + extrahepatic	4.21 (1.82–9.69)	**< 0.001**	11.17 (4.03–30.98)	**< 0.001**	6.85 (2.50-18.71)	**< 0.001**	7.70 (2.81–21.07)	**< 0.001**
Performance status								
0–1	1		1		1		1	
2–4	3.10 (1.53–6.31	**0.002**	4.45 (1.92–10.30)	**< 0.001**	2.94 (1.18–7.34)	.**020**	3.14 (1.29–7.68)	**0.012**
Resection of metastatic lesion(s)								
No	1		1		1		1	
Yes	0.57 (0.32–1.01)	0.053	1.03 (0.50–2.13)	0.937	0.91 (0.44–1.91)	0.818	0.91 (0.44–1.90)	0.808
First-line ipi + nivo^a^								
No	1		STRATA		STRATA		STRATA	
Yes	0.65 (0.4–1.01)	**0.057**	STRATA		STRATA		STRATA	
Whole-body SUV_max_								
SUVmax < 10.1	1							
SUVmax > 10.1	1.72 (1.16–2.56)	**0.008**						
Log10(SUVmax)			1.90 (1.19–3.05)	.**007**				
Whole-body MTV (cm^3^)								
MTV < 14.6	1							
MTV > 14.6	2.09 (1.40–3.13)	**< 0.001**						
Sqrt(MTV)	1.12 (1.09–1.16)	**< 0.001**			1.17 (1.10–1.25)	**< 0.001**		
Whole-body TLG								
TLG < 85.5	1							
TLG > 85.5	2.08 (1.39–3.09)	**< 0.001**						
Sqrt(TLG)	1.04 (1.03–1.05)	**< 0.001**					1.06 (1.04–1.09)	**< 0.001**

Linearity test indicated transformation of MTV and TLG with square root (sqrt) and with log10 for SUVmax. The covariate ipi + nivo treatment was non-proportional and was included as strata in the multivariate Cox regression analyses.

a First-line treatment with the combination of ipilimumab and nivolumab.

AJCC = American Joint Committee on Cancer, ipi + nivo = ipilimumab and nivolumab.

Three multivariate Cox regression analyses were performed for log10-SUV_max_, sqrt-MTV, and sqrt-TLG, respectively. Log10-SUV_max_, sqrt-MTV, and sqrt-TLG stayed significant in each of the multivariate Cox regression analyses along with the metastatic pattern and the performance status (Table 2). The AJCC stage turned insignificant in the multivariate Cox regression analyses with sqrt-MTV and sqrt-TLG, respectively, whereas the AJCC stage stayed significant in the log10-SUV_max_ multivariate Cox.

In the Cox regression sub-analyses of the 97 patients with liver metastases, log10-liver-SUV_max_ (HR 1.89 (95% CI 1.18–3.02), *p* = 0.008), sqrt-liver-MTV (HR 1.16 (95% CI 1.10–1.23), *p* < 0.001), and sqrt-liver-TLG (HR 1.06 (95% CI 1.04–1.09), *p* < 0.001), were accordingly only significant in the multivariate Cox analysis with log10-liver-SUV_max_ (Supplementary Table S1).

### Time-dependent ROC curves

**Fig. 2:**
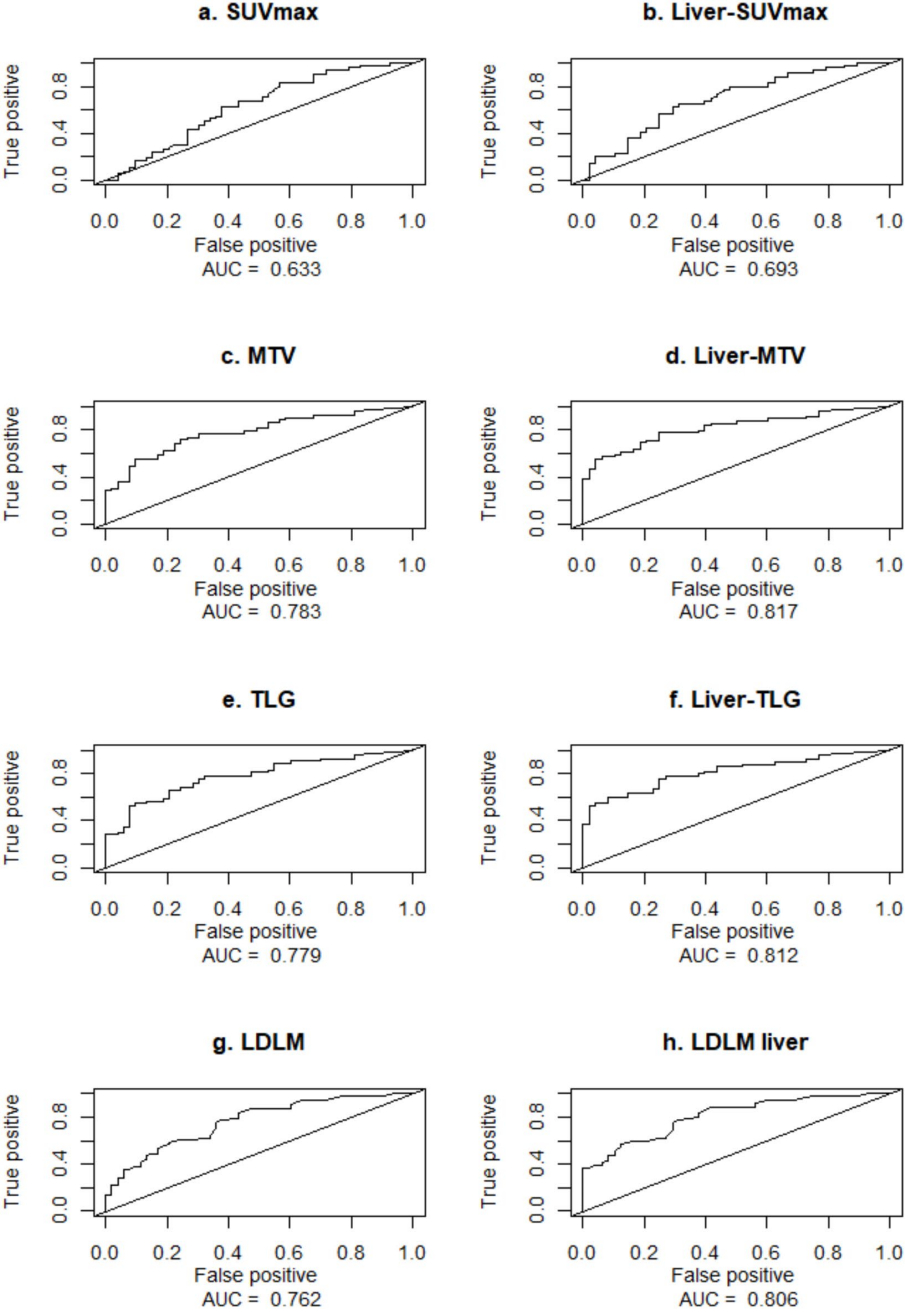
Time-dependent receiver operating characteristics (ROC) curves for 1-year survival prediction with area under the curve (AUC) for **(a)** whole-body SUV_max_, **(b)** liver-SUV_max_, **(c)** whole-body MTV, **(d)** liver-MTV, **(e)** whole-body TLG, **(f)** liver-TLG, **(g)** LDLM, and **(h)** LDLM in the liver. Overall, the AUCs for the MTV and TLG values are highest, followed by LDLM. The AUCs of the liver parameters are higher than the whole-body parameters, with liver-TLG and liver-MTV demonstrating the highest AUC of 0.812 and 0.817, respectively. This indicates that the SUV_max_, MTV, TLG, and LDLM of only the liver are more precise predictors of survival than the whole-body parameters in patients with liver metastases. LDLM = largest diameter of the largest metastatic lesion, MTV = Metabolic Tumor Volume, TLG = Total Lesion Glycolysis, SUVmax = Maximal Standardized Uptake Value.

The time-dependent 1-year survival Receiver Operating Characteristics (ROC) curves for both MTV (Area Under the ROC Curve (AUC) = 0.78, Fig. 2c), TLG (AUC = 0.78, Fig. 2e), and LDLM (AUC = 0.76, Fig. 2g) showed a good discriminative ability to predict the 1-year survival. The 1-year ROC for SUV_max_ indicated a poorer discriminative ability (AUC of 0.63, Fig. 2a).

For the subset of 97 patients with liver metastases, the 1-year ROC curves for tumor burden in the liver only showed corresponding, but slightly higher, AUC values: liver-SUV_max_ (AUC = 0.69, Fig. 2b), liver-MTV (AUC = 0.81, Fig. 2d), liver-TLG (AUC = 0.812, Fig. 2f), and LDLM in the liver (AUC = 0.806, Fig. 2h).

### Positive predictive value of 1-year survival

**Fig. 3:**
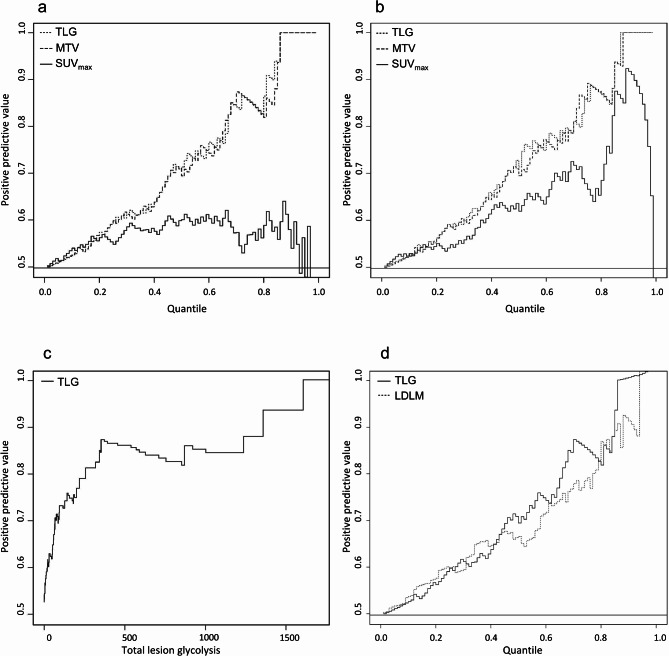
1-year positive predictive value (PPV) analyses in 106 patients with metastatic posterior uveal melanoma. **(a)** Unadjusted 1-year PPV for, respectively, whole-body Total Lesion Glycolysis (TLG) (dotted line), whole-body Metabolic Tumor Volume (MTV) (dashed line), and whole-body Maximal Standardized Uptake Value (SUV_max_) (solid line) as a function of quantiles of whole-body TLG, whole-body MTV, and whole-body SUV_max_. The lines for TLG and MTV increase steadily towards the upper right corner of the plot, indicating that the probability of death increases with increasing values of TLG and MTV. **(b)** 1-year PPV for whole-body TLG (dotted line), whole-body MTV (dashed line), and whole-body SUV_max_ (solid line) adjusted for AJCC. The PPV for SUV_max_ improves when adjusting for AJCC, whereas the lines for MTV and TLG do not change. This indicates that the LDLM contributes an additive effect only to the SUV_max_. **(c)** 1-year PPV of whole-body TLG as a function of the absolute values of whole-body TLG. The line reaches PPV(1.0) at a whole-body TLG of approximately 1600. **(d)** 1-year PPV comparing unadjusted whole-body TLG (solid line) and the largest diameter of the largest metastatic lesion (LDLM) (dotted line). Both lines have a sharp and steady increase towards the upper right corner of the plot, indicating, that the PPV of TLG and LDLM are comparable.

The 1-year univariate positive predictive value (PPV) for SUV_max_, MTV, and TLG were calculated (Fig. 3a). The lines for TLG and MTV constantly increase from the starting point towards a PPV of 1.0, as the quantiles increase towards the highest, most extreme values, indicating a good prognostic value. The line for SUV_max_ shortly increases and then decreases, indicating a poor PPV. When adjusting for the AJCC stage, the 1-year PPV for TLG and MTV were unaffected (Fig. 3b), but the prognostic value of SUV_max_ improved markedly, though still inferior to MTV and TLG. An MTV value of approximately 210 and a TLG value of approximately 1600 equaled a PPV of 1.0 (graph for TLG shown in Fig. 3c), which means that the likelihood of death within one year was 100% when MTV was higher than 210, and when TLG was higher than 1600. The likelihood of death within one year was 80% when MTV was higher than approximately 50 and TLG was higher than approximately 260. Figure 3d compares the 1-year PPV for LDLM to that of TLG. Both lines sharply increase towards the highest and most extreme values of TLG and LDLM, indicating a good prognostic value for both variables.

Regarding the subset of patients with metastasis to the liver, the univariate PPV analysis showed accordingly that liver-MTV and liver-TLG were equally good predictors of 1-year survival, whereas liver-SUV_max_ had a poorer prognostic value (Supplementary Fig. S2a). When adjusting for AJCC, the 1-year PPV for liver-SUV_max_ was now comparable to liver-MTV and liver-TLG, whereas liver-TLG and liver-MTV did not change (Supplementary Fig. S2b). The likelihood of death within one year was 100% when liver-MTV was approximately 130 and liver-TLG was approximately 870 (graph for liver-TLG shown in Supplementary Fig. S2c). The likelihood of death within one year was 80% when liver-MTV was approximately 30 and liver-TLG was approximately 140. Supplementary Fig. S2d compares the 1-year unadjusted PPV for liver-LDLM and liver-TLG. Both lines show a steady increase toward higher and more extreme values of liver-TLG and liver LDLM, indicating a good prognostic value for both variables.

## Discussion

In this study, we showed that the ^18^F-FDG PET/CT-derived metabolic activity measurements of tumor tissue SUV_max_, MTV, and TLG were correlated to overall survival in patients with metastatic PUM, as high values above the median were associated with shorter survival. All three parameters were identified as independent predictors of survival in the univariate and multivariate Cox regressions and were superior to AJCC, though the nonlinearity of SUV_max_, MTV, and TLG demanded a transformation of the variables. The 1-year ROC curve analysis and the 1-year PPV analysis showed that MTV, TLG, and LDLM were accurate predictors of 1-year survival, as high levels of MTV, TLG, and LDLM were associated with a higher risk of death at one year. SUV_max_ demonstrated an inferior prognostic effect. Interestingly, TLG and MTV performed equally well in the 1-year ROC analysis and the 1-year PPV analysis, which suggests that the metabolic active tumor volume, rather than the intensity of the metabolic activity, is most important for survival. The adjusted 1-year PPV analysis showed no overall change in PPV for MTV or TLG, whereas the PPV of SUV_max_ increased significantly when adjusting for AJCC. This is not surprising, as the AJCC/LDLM roughly reflects the tumor size and thereby contributes an additive effect only to the SUV_max_.

The LDLM was not applicable for the multivariate Cox regression analysis, but interestingly the 1-year PPV and 1-year ROC AUC for LDLM were comparable to TLG and MTV. LDLM is easy to obtain but is a less objective parameter than MTV and TLG. Even though it only reflects the size of the largest metastasis it quite accurately predicted the 1-year survival. The AJCC stage did not stay significant in the multivariate Cox regression analyses, which indicates that the prognostic value of LDLM decreases when categorized into the three AJCC categories.

The majority of the patients in this cohort presented with liver metastases (92%), which is in line with previous studies^5,6^. Interestingly, the sub-analyses of the patients with liver metastases showed that the regional liver-SUV_max_, liver-MTV, and liver-TLG were slightly better predictors of the 1-year survival than the whole-body measurements, as the AUC was higher. The 1-year PPV for liver-MTV and liver-TLG was also comparable to that of whole-body MTV and TLG, and the 1-year unadjusted and adjusted PPV analysis for liver-SUV_max_ was superior to that of whole-body SUV_max_. This indicates that for patients with liver metastases, the hepatic parameters are good and maybe even better predictors of the 1-year survival than the whole-body parameters, and it raises the hypothesis that the mortality primarily is driven by the hepatic tumor burden in these patients. However, the multivariate HR’s of the regional liver parameters and the whole-body parameters were similar.

To the best of our knowledge, the present study is the first to compare the predictive value of whole-body SUV_max_, MTV, and TLG to that of the liver, and to compare it to the LDLM. Carpio et al.^22^ previously investigated the prognostic role of SUV_max_ in metastatic uveal melanoma liver lesions and demonstrated that increased SUV_max_ was associated with poor survival and higher HR for death. In a treatment study of yttrium-90 microsphere brachytherapy for inoperable liver metastases from uveal melanoma, Eldredge-Hindy et al. reported that increasing levels of pretreatment MTV and TLG of liver metastases from uveal melanoma were associated with shorter survival, whereas SUV_max_ was not associated with survival outcomes^23^. Neither MTV nor TLG is routinely obtained from ^18^F-FDG PET/CT, as it requires time-consuming outlining of all tumors. However, when liver metastases are present it might be sufficient to only analyze the liver, as the liver-MTV and liver-TLG were found to be the best predictors of 1-year survival. Currently, the measurement of LDLM is straightforward to use, as it can be obtained from liver ultrasonography, CT, or MRI^25^. As a result, staging patients according to AJCC/LDLM continues to be the preferred method. However, implementation of automatized calculations of SUV_max_, MTV, and TLG is ongoing and could be applied in future clinical practice, supported by the use of radiomics through artificial intelligence^26–28^. Various prognostic multifactor models have been proposed for predicting outcomes in metastatic PUM^29–31^, incorporating not only tumor size but also factors such as lever enzyme levels and performance status. It would be interesting to explore whether incorporating TLG and/or MTV could enhance the predictive accuracy of prognostic models in the future.

TLG and MTV have proved to be independent prognostic factors in patients with metastatic cutaneous melanoma with the potential to improve risk-stratification of the patients^18,32,33^. Son et al. have also proposed that MTV and/or TLG of primary cutaneous melanoma could guide a more personalized follow-up program, with close surveillance of patients with high values of MTV and/or TLG^34^. Ito et al. have found that MTV obtained before the start of systemic treatment is strongly associated with survival in patients with metastatic cutaneous melanoma, and the authors suggest including the MTV level, when stratifying the patients into clinical trials, or when selecting eligible patients for experimental therapy studies^32^. Our study suggests that the use of MTV and TLG likewise could be applied in the management of metastatic PUM.

The physiological glucose uptake in the liver is high, resulting in a so-called “mottled” background, that makes it difficult to distinguish between normal liver tissue and malignant tumors^35^. The SUV_max_ of normal liver tissue ranges between 3.0 and 4.0^16^. As the vast majority of the patients with metastatic PUM present with liver metastases, we chose a fixed absolute SUV threshold of 4.0. There is no consensus about the best segmentation method, but when it comes to survival prediction, fixed absolute thresholds have been found to have a better prognostic value than a fixed-relative threshold investigated in other cancers^17,36^. Nevertheless, there are limitations related to choosing a fixed absolute threshold. With a relatively high fixed threshold of SUV 4.0, we might miss some lesions with low metabolism and thereby underestimate the tumor volume^17^. Contrarily, a fixed absolute threshold might introduce a spillover effect in tumors with high SUV, resulting in an overestimation of the volume^17^.

We acknowledge that there are other inherent limitations in this study. The study is a retrospective study, which might introduce a selection bias, as PET/CT scans are offered to Danish patients with metastatic PUM, who are considered eligible for treatment. Due to the rarity of metastatic PUM, we included PET/CT scans performed on various scanners from multiple centers throughout Denmark spanning two decades, but the majority of scans (79%) were performed at Copenhagen University Hospital – Rigshospitalet on Siemens Biograph scanners, see Supplementary Table S2. Variations in scanner resolution and reconstruction parameters potentially introduce a bias in estimates of SUV_max_, and to a minor extent TLG and MTV^37^. Despite the heterogeneity of data and the lack of harmonization strategies to standardize data across different centers, we showed that MTV and TLG are good predictors of survival in patients with metastatic PUM, underlining the promising role that these parameters could play in patient risk assessment.

## Methods

### Patients

From the Danish research database Copenhagen Epidemiological Uveal Melanoma Study (COEUS)^38^, 252 patients with primary PUM (choroidal and ciliary body melanoma) diagnosed from 2000 to 2020 and with subsequent development of metastatic disease until the end of 2022 were identified. One hundred and eleven patients (44%) had undergone ^18^F-FDG PET/CT imaging within 5 weeks from the date of metastatic diagnosis, of which two patients were excluded due to poor quality of the ^18^F-FDG PET images, and three patients were excluded due to other cancer diagnoses. All 106 retrospectively included patients had been treated for primary PUM at the Department of Ophthalmology at Copenhagen University Hospital - Rigshospitalet. The included cohort of 106 patients is a subset of a previously published cohort of 178 patients with metastatic PUM^39^. A flowchart of the inclusion and exclusion process is shown in Supplementary Fig. S1.

### Ethics

The study followed the Declaration of Helsinki and was approved by the Regional Ethics Committee of the Capitol Region of Denmark, who also granted a waiver of informed consent as the vast majority of patients included in this study were deceased (reference H-21015415, 8th of July, 2021). When permission to access patient data has been granted, Danish law allows the transfer of the permitted data between hospitals. All quantitative measurements from obtained scans were assessed at the Department of Clinical Physiology and Nuclear Medicine at Copenhagen University Hospital – Rigshospitalet, and the data were then pseudo anonymized before further statistical analysis.

### Clinical data

The following clinical data were retrieved from the medical records, the COEUS database, and the Danish Metastatic Melanoma Database (DAMMED)^40^: age, sex, AJCC stage IV (M1a, M1b, M1c), LDLM on CT or MRI, Eastern Cooperative Oncology Group (ECOG) performance status, metastatic pattern: (a) exclusively hepatic metastases (hepatic pattern), (b) both hepatic and extrahepatic metastases (hepatic-extrahepatic pattern), and (c) exclusively extrahepatic metastases (extrahepatic pattern), whether patients underwent resection of metastatic lesions or not, and whether patients were treated with first-line combination of ipilimumab and nivolumab or not. Ipilimumab and nivolumab were introduced as first-line treatment in Denmark in June 2016. Whether patients were treated with this combination regimen or not was chosen as the treatment covariate as this is the preferred oncological treatment available for Danish patients, which has shown some but modest effect in metastatic uveal melanoma^41,42^. The vital status of the patients was retrieved on December 31, 2023, allowing a minimum of 1 year of follow-up from the date of metastatic diagnosis. Six patients were still alive at the end of follow-up, and metastatic PUM was the cause of death in all deceased patients.

### 18F-FDG PET-CT imaging

All included patients underwent clinical ^18^F-FDG PET/CT imaging covering at least the region from the neck to the mid-thigh. Instructions for fasting varied between sites, with a minimum of 4 h of recommended fasting. The median ^18^F-FDG dose was 3.97 Mbq/kg (IQR 3.80-4.00 Mbq/kg). The majority (*n* = 85) of the included scans were performed at Copenhagen University Hospital – Rigshospitalet, and the remaining (*n* = 21) were performed at 9 different local hospitals in Denmark. The scans were performed with various dedicated PET/CT scanners and reconstruction algorithms: Siemens Biograph (*n* = 95) (Vision, mCT, or TruePoint) reconstructed with OSEM, GE Healthcare Discovery (*n* = 6) (LS, 710, MI, 690, or RX) reconstructed with OSEM or Q.Clear, and Philips Gemini TF (*n* = 3) reconstructed with BLOB. See Supplementary Table S2 for details regarding specifications of the imaging equipment and reconstruction algorithms used, including matrix size, pixel size, Gaussian filter, and number of iterations and subsets. No harmonization strategies were applied to standardize data across different centers.

### Image analysis

**Fig. 4:**
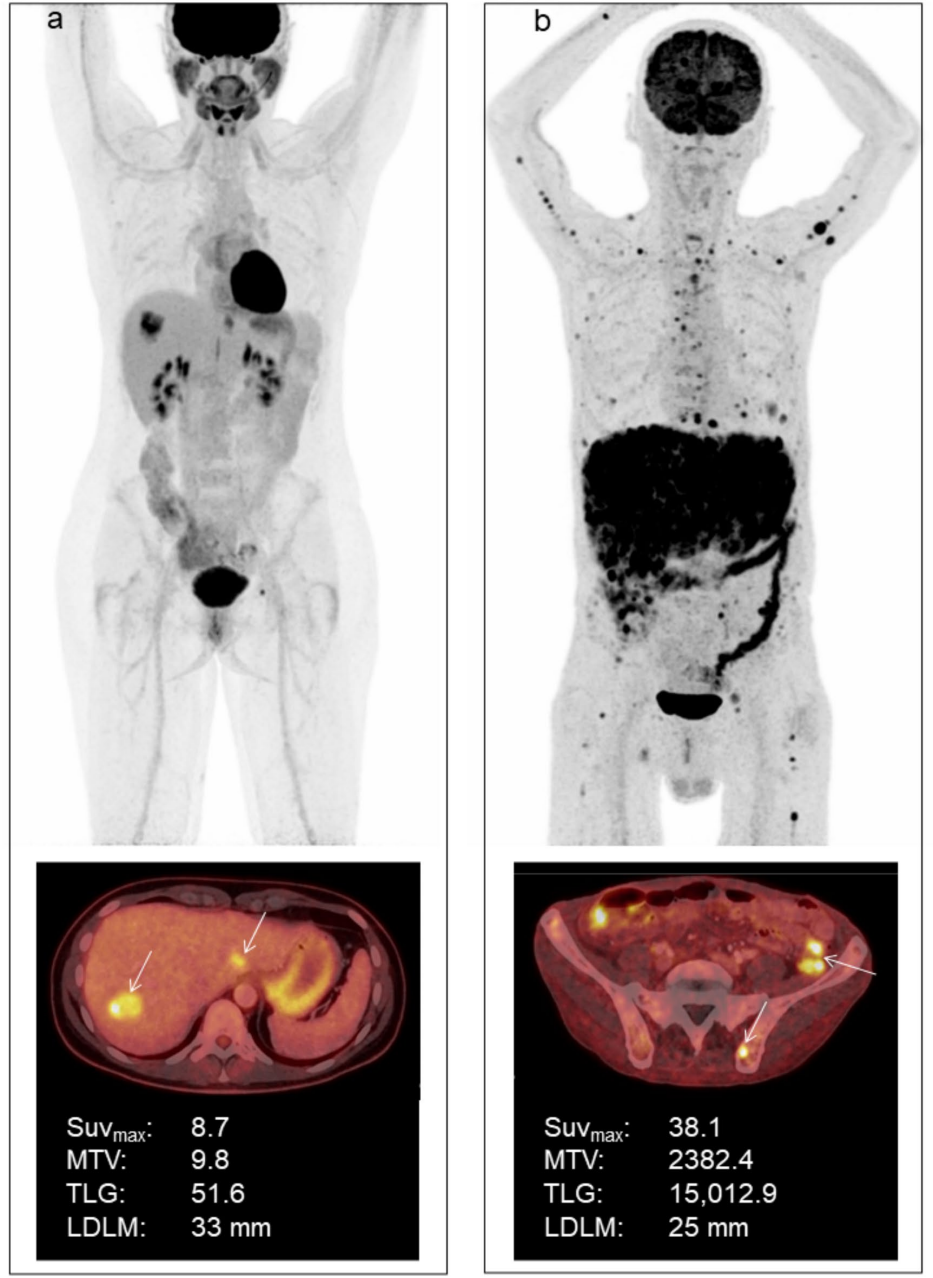
(a) Whole-body ^18^F-FDG PET/CT image of a female patient with two liver metastases from posterior uveal melanoma with an overall survival of 18 months. The two liver metastases are marked with arrows on the axial image; **b)** Whole-body ^18^F-FDG PET/CT image of a male patient with posterior uveal melanoma metastasized to various organs, including liver, lung, pleura, bone, muscle, lymph nodes, and the thyroid, and with an overall survival of 1 month. The axial image shows a metastasis in the right iliac bone and two carcinosis elements (arrows).

LDLM was measured on an axial image of liver MRI by author and radiologist CWB. If a liver MRI was unavailable or not performed, the LDLM was measured on an axial image of the CT correlate as part of the clinical examination. All clinically described metastatic lesions were outlined using a fixed SUV threshold of 4.0, which made it possible to discriminate tumor tissue from healthy metabolically active liver tissue^16^. Delineations of all lesions were performed by physician TGH and verified by physician and specialist in nuclear medicine KM. SUV_max_, MTV, and TLG for each delineated lesion were measured, and whole-body MTV and TLG were subsequently summed for each patient. In three patients presenting with metastatic disease at primary diagnosis, the metabolic activity of the primary eye tumor was included in the analysis. Among patients with liver metastases, liver-SUV_max_, liver-MTV, and liver-TLG for all liver lesions were obtained separately. In 12 cases with ^18^F-FDG-uptake < SUV 4.00 in the metastatic lesions, SUV_max_ was determined using the co-registered CT or MRI correlate to identify the metastatic lesions. Images of two representative patients are illustrated in Fig. 4.

### Statistics

Overall survival was defined as survival from the date of the ^18^F-FDG PET-CT imaging to the date of death or last follow-up. Survival probabilities were visualized using Kaplan-Meier curves for the variables SUV_max_, MTV, and TLG, where the patients were dichotomized into high and low groups using the median values of each parameter, and differences in survival were tested with the log-rank test. Differences in survival were also tested with the Cox proportional hazard model, with covariates chosen based on clinical relevance. Cumulative martingale residuals and cumulative score process tests were used to evaluate the assumption of proportional hazards. Covariates with non-proportionality were included as strata in the multivariate Cox regression.

As the effect of the dichotomized MTV and TLG were non-proportional, the multivariate Cox regressions were performed with SUV_max,_ MTV, and TLG, respectively, as continuous variables only. The AJCC was included in the Cox regressions, while the continuous variable LDLM was non-proportional and therefore not included. To assess the linearity of the effect of the continuous variables, residuals from the Cox model were plotted against the covariate and the transformed forms of the covariate (natural logarithm, log10, and square root (sqrt)). The linearity test showed that SUV_max_ had to be log10-transformed, whereas MTV and TLG had to be sqrt-transformed.

Time-dependent ROC curves for the continuous variables SUV_max_, MTV, TLG, and LDLM were conducted, with time fixed to 1 year. AUC was used to evaluate the discriminative ability of the variables.

To assess how well SUV_max_, MTV, TLG, and LDLM identify the patients who will experience the event within one year (event = death yes/no), the time-dependent PPV was calculated^43^. PPV is defined by PPV(q) = P(T < t | X > q), where T denotes the survival time, X is the biomarker of interest (SUV_max_, MTV, TLG, or LDLM), q is the corresponding q-quantile that is varied, and t denotes the time, which was fixed to 1 year. Hence, as an example, PPV(0.9) is the probability of dying within the first year of follow-up for a patient with a biomarker value above the 90% quantile. The PPV value for the 0% quantile corresponds to the overall 1-year risk, so the PPV curve for any biomarker starts there, and a good biomarker has a (sharply) increasing curve towards 1.0.

A *p*-value < 0.05 was considered statistically significant. Statistical analysis was performed using Rstudio (version 2023.06.0) (Rstudio Team, 2023) with the packages: ‘survival’ (version 3.5.5), ‘timereg’ (version 2.0.5), ‘survminer’ (version 0.4.9), ‘survivalROC’ (version 1.0.3.1). The PPV analysis was coded directly in Rstudio by author and biostatistician TM, without the use of any external packages.

## Conclusion

MTV and TLG were identified as equally good predictors of survival in patients with metastatic PUM and they were both superior to the currently used AJCC staging system, which is based on LDLM categorization. This emphasizes the clinical relevance of metabolic tumor burden obtained from ^18^F-FDG PET/CT imaging in patients with metastatic PUM and suggests that the quantitative measurements of the metabolic tumor burden could play an important prognostic role. For 1-year survival specifically, the prognostic value of LDLM as a continuous variable was surprisingly comparable to MTV and TLG.

For patients with liver metastases, the regional liver-MTV and liver-TLG of only the liver lesions proved to be at least as good predictors of survival as the whole-body parameters, which might indicate that the mortality primarily is driven by the hepatic tumor burden in this subset of patients.

## Electronic supplementary material

Below is the link to the electronic supplementary material.

Supplementary Material 1

## Data Availability

The patient data included in this study cannot be completely anonymized and consequently cannot be made publicly accessible following the European General Data Protection Regulation (GDPR). For inquiries about the data, please contact Professor J.F.K (jens.folke.kiilgaard@regionh.dk).
