# Supplementary material for: 18F-FDG PET/CT assessment of metabolic tumor burden predicts survival in patients with metastatic posterior uveal melanoma
Source: Sci Rep. 2025 Feb 3;15:4110. doi: 10.1038/s41598-025-88625-w (PMC11790917; doi:10.1038/s41598-025-88625-w)
Supplement: Supplementary file 1 — Supplementary Material 1 [file 41598_2025_88625_MOESM1_ESM.docx]

# Supplementary information

**Title:** ^18^F-FDG PET/CT assessment of metabolic tumor burden predicts survival in patients with metastatic posterior uveal melanoma

**Journal:** Scientific Reports

**Authors:** Tine Gadegaard Hindso^1^, Torben Martinussen^2^, Camilla Wium Bjerrum^3^, Sune Høgild Keller^4^, Annika Loft^4^, Mette Bagger Sjøl^1^, Kristoffer Nissen^1^, Carsten Faber^1^, Marco Donia^5^, Inge Marie Svane^5^, Eva Ellebaek^5^, Steffen Heegaard^1,6^, Jens Folke Kiilgaard^1^, Karine Madsen^4^

**Affiliations:**

1: Department of Ophthalmology, Copenhagen University Hospital – Rigshospitalet, Blegdamsvej 9, 2100 Copenhagen Ø, Denmark

2: Department of Biostatistics, University of Copenhagen, Øster Farimagsgade 5, 1014 Copenhagen K, Denmark

3: Department of Radiology, Copenhagen University Hospital – Rigshospitalet, Blegdamsvej 9, 2100 Copenhagen Ø, Denmark, Copenhagen, Denmark

4: Department of Clinical Physiology and Nuclear Medicine, Copenhagen University Hospital – Rigshospitalet, Blegdamsvej 9, 2100 Copenhagen Ø, Denmark

5: National Center for Cancer Immune Therapy (CCIT-DK), Department of Oncology, Copenhagen University Hospital – Herlev and Gentofte, Borgmester Ib Juuls Vej 13, 2730 Herlev, Denmark

6: Department of Pathology, Copenhagen University Hospital – Rigshospitalet, Blegdamsvej 9, 2100 Copenhagen Ø, Denmark

**Corresponding author:** Tine Gadegaard Hindso, mail: tinegadegaard@gmail.com

**Table of contents:**

Supplemental Figures……………………………………………………………………………………………………2

Fig. S1………………………………………………………………………………………………….2

Fig. S2………………………………………………………………………………………………….3

Supplemental Tables

Table S1…………………………………………………………………………………………………4

Table S2…………………………………………………………………………………………………5

**
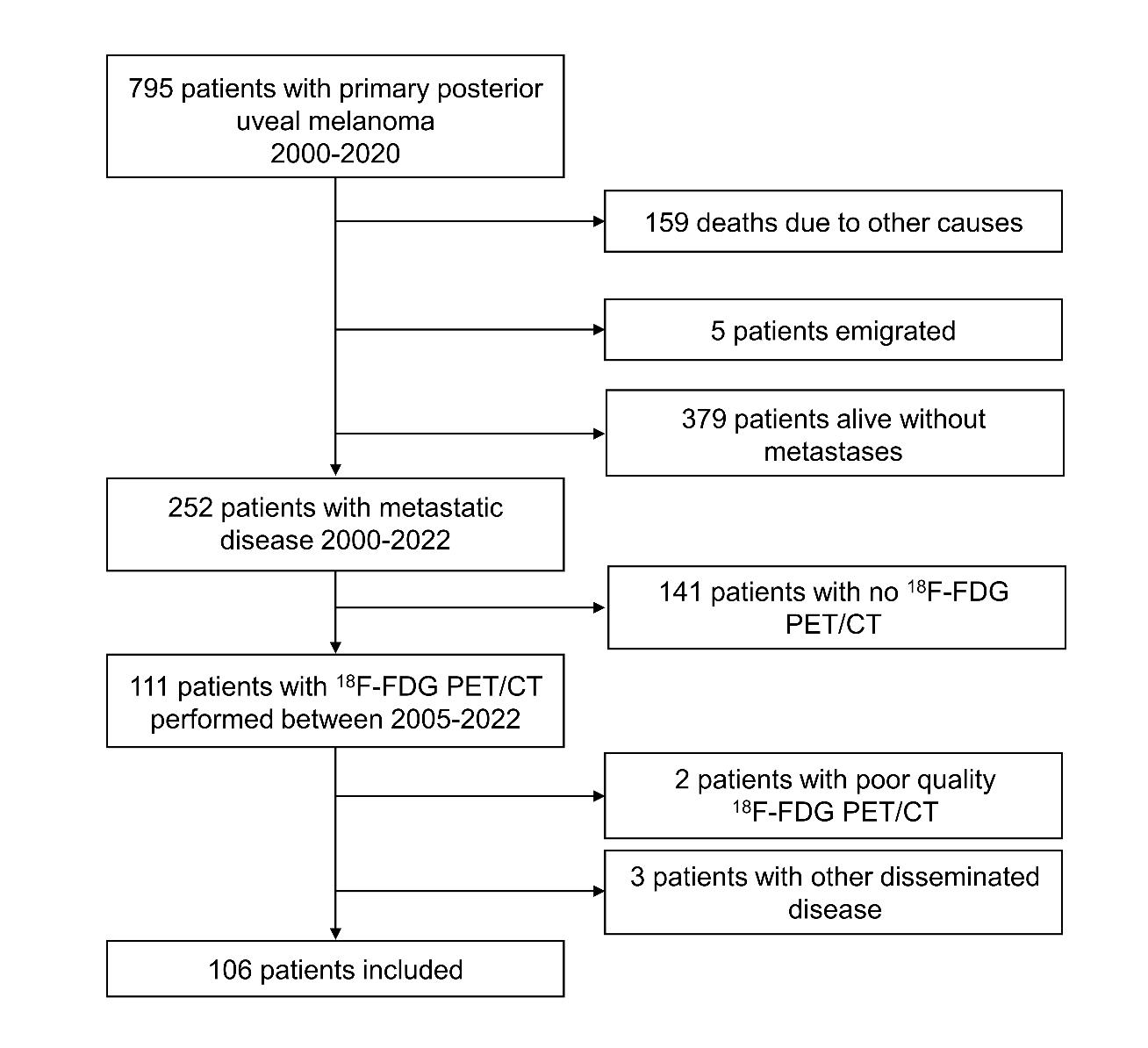
**

**Figure. S1.** Flow chart of the process of inclusion and exclusion of patients in the study.


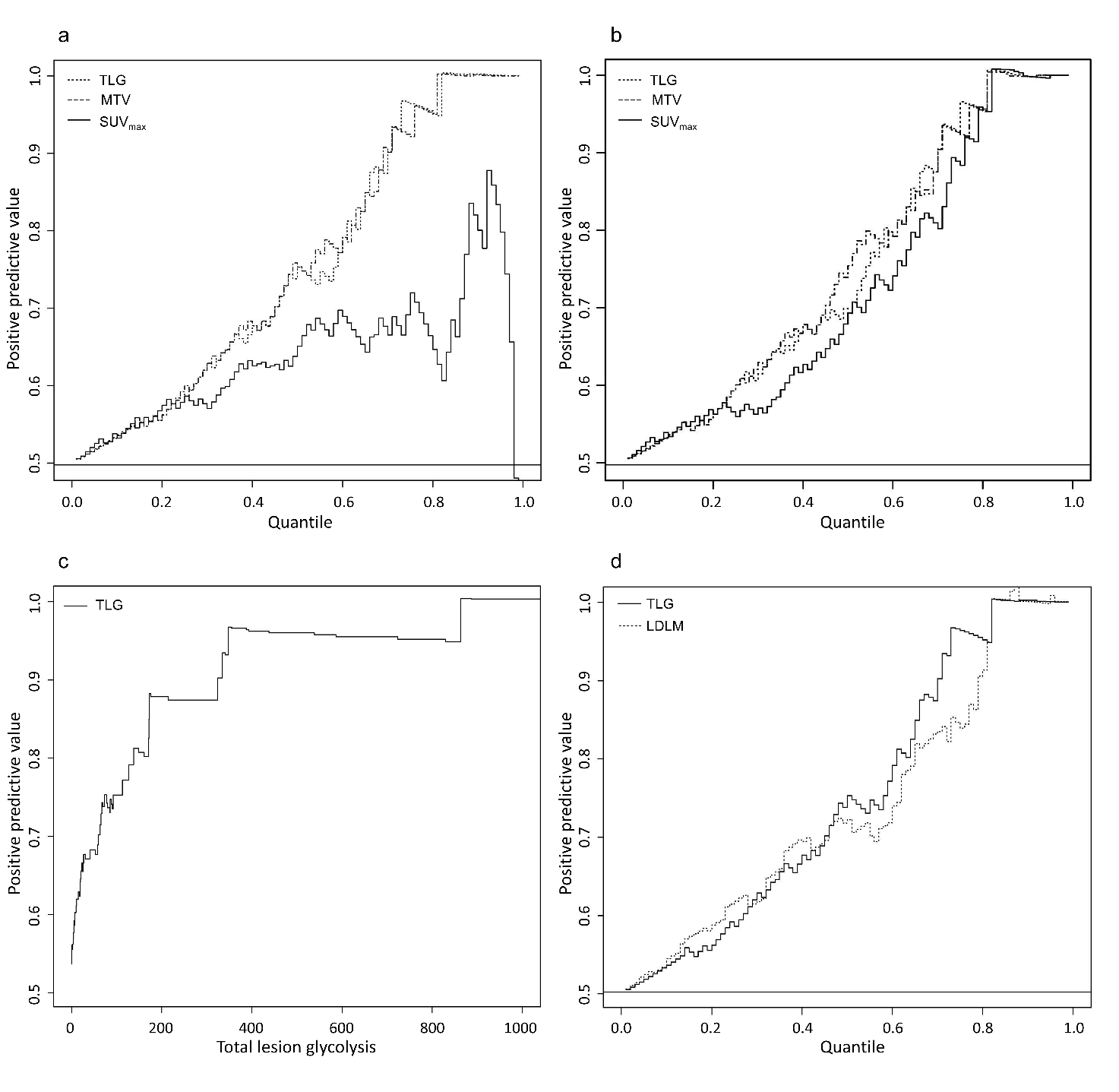


**Figure. S2. (a)** Unadjusted 1-year positive predictive value (PPV) for liver-TLG (dotted line), liver-MTV (dashed line), and liver-SUV_max_ (solid line) as a function of the quantiles of liver-TLG, liver-MTV, and liver-SUV_max_, respectively. The sharp and steady increase in the liver-MTV and liver-TLG lines indicates a good PPV. **(b)** 1-year PPV for liver-TLG (dotted line), liver-MTV (dashed line), and liver-SUV_max_ (solid line), adjusted for AJCC shows an improve in PPV for SUV_max_. The PPV for liver-SUV_max_ is now equal to liver-MTV and liver-TLG **(c)** 1-year PPV of liver-TLG as a function of the absolute values of liver-TLG. The line reaches PPV(1.0) at a liver-TLG of approximately 870, and **(d)** 1-year PPV for liver-TLG (solid line) and LDLM in the liver (dotted line) shows that the PPV’s are comparable.

|  | **Univariate Cox model** | | **Multivariate Cox model**  **with liver-SUVmax** | | **Multivariate Cox model**  **with liver-MTV** | | **Multivariate Cox model**  **with liver-TLG** | |  |
| --- | --- | --- | --- | --- | --- | --- | --- | --- | --- |
|  | **HR (95% CI)** | ***p*** | **HR (95% CI)** | ***p*** | **HR (95% CI)** | ***p*** | **HR (95% CI)** | ***p*** |  |
| **Sex** |  |  |  |  |  |  |  |  |  |
| Women | 1 |  | 1 |  | 1 |  | 1 |  |  |
| Men | 1.01 (0.67-1.54) | .947 | 1.22 (0.72-2.06) | .461 | 1.06 (0.64-1.75) | .828 | 1.12 (0.69-1.83) | .653 |  |
| **Age** |  |  |  |  |  |  |  |  |  |
| Under 60 years of age | 1 |  | 1 |  | 1 |  | 1 |  |  |
| Over 60 years of age | 1.46 (0.01-2.37) | .122 | 1.23 (0.71-2.11) | .460 | 1.24 (0.71-2.17) | .456 | 1.44 (0.82-2.52) | .202 |  |
| **AJCC stage IV** |  |  |  |  |  |  |  |  |  |
| M1a | 1 |  | 1 |  | 1 |  | 1 |  |  |
| M1b | 1.71 (1.08-2.69) | **.021** | 1.50 (0.87-2.58) | .147 | 0.94 (0.52-1.70) | .848 | 0.96 (0.54-1.69) | .882 |  |
| M1c | 12.10 (5.73-25.55) | **< .001** | 14.86 (3.87-57.15) | **< .001** | 2.32 (0.42-12.95) | .338 | 1.84 (0.51-6.63) | .354 |  |
| **Metastatic pattern** |  |  |  |  |  |  |  |  |  |
| Hepatic | 1 |  | 1 |  | 1 |  | 1 |  |  |
| Hepatic + extrahepatic | 2.46 (1.56-3.89) | **< .001** | 2 02 (1.16-3.51) | **.012** | 1.82 (1.02-3.23) | **.040** | 1.67 (0.94-2.97) | .081 |  |
| **Performance status** |  |  |  |  |  |  |  |  |  |
| 0-1 | 1 |  | 1 |  | 1 |  | 1 |  |  |
| 2-4 |  |  | 2.15 (0.74-6.25) | .158 | 1.35 (0.42-4.38) | 0.613 | 1.57 (0.56-4.40) | .390 |  |
| **Resection of metastatic lesion(s)** |  |  |  |  |  |  |  |  |  |
| No | 1 |  | 1 |  | 1 |  | 1 |  |  |
| Yes | 0.48 (0.27-0.87) | **.015** | 0.87 (0.42-1.80) | .708 | 0.83 (0.40-1.74) | .635 | 0.75 (0.37-1.56) | .446 |  |
| **First-line ipi+nivo^a^** |  |  |  |  |  |  |  |  |  |
| No | 1 |  | STRATA |  | STRATA |  | STRATA |  |  |
| Yes | 0.61 (0.39-0.96) | **.033** | STRATA |  | STRATA |  | STRATA |  |  |
| **Liver-SUV_max_** |  |  |  |  |  |  |  |  |  |
| SUVmax < 8.9 | 1 |  |  |  |  |  |  |  |  |
| SUVmax > 8.9 | 2.01 (1.32-3.05) | **.001** |  |  |  |  |  |  |  |
| Log10(SUVmax) | 1.88 (1.32-2.69) | **<.001** | 1.89 (1.18-3.02) | **.008** |  |  |  |  |  |
| **Liver-MTV (cm^3^)** |  |  |  |  |  |  |  |  |  |
| MTV < 13.8 | 1 |  |  |  |  |  |  |  |  |
| MTV > 13.8 | 2.47 (1.61-3.77) | **<.001** |  |  |  |  |  |  |  |
| Sqrt(MTV) | 1.15 (1.11-1.19) | **<.001** |  |  | 1.16 (1.10-1.23) | **<.001** |  |  |  |
| **Liver-TLG** |  |  |  |  |  |  |  |  |  |
| TLG < 73.6 | 1 |  |  |  |  |  |  |  | |
| TLG > 73.6 | 2.31 (1.52-3.51) | **<.001** |  |  |  |  |  |  | |
| Sqrt(TLG) | 1.05 (1.04-1.07) | **<.001** |  |  |  |  | 1.06 (1.04-1.09) | **<.001** | |

**Table S1.** Univariate and multivariate Cox proportional hazard regression models for overall survival from date of baseline PET-CT to death or last follow-up in 97 metastatic liver lesions from posterior uveal melanoma patients. Three multivariate Cox regressions was performed with, respectively, liver-SUV_max_, liver-MTV, and liver-TLG. Linearity test indicated transformation of liver-MTV and liver-TLG with square root and transformation with log10 for SUVmax. The non-proportional covariate treatment with ipilimumab and nivolumab was included as strata in the multivariate Cox regression analyses.

^a^ First-line treatment with the combination of ipilimumab and nivolumab.

Abbreviations: AJCC = American Joint Committee on Cancer; ipi+nivo = ipilimumab and nivolumab.

| No. of subjects | Hospital | Scanner | Matrix size | XY-pixel size (mm) | Gaussian  reconstruction  filter (mm) | Reconstruction method | Iterations and subsets |
| --- | --- | --- | --- | --- | --- | --- | --- |
| 4 | A | Siemens Biograph VisionQuadra | 440x440 | 1.65x1.65 | 2.00 | OSEM3D+PSF+TOF | 4i5s |
| 13 | A | Siemens Biograph Vision | 440x440 | 1.65x1.65 | 2.00 | OSEM3D+PSF+TOF | 4i5s |
| 13 | A | Siemens Biograph mCT | 400x400 | 2.04x2.04 | 2.50 | OSEM3D+PSF+TOF | 2i21s |
| 24 | A | Siemens Biograph mCT | 400x400 | 2.04x2.04 | 2.00 | OSEM3D+PSF+TOF | 2i21s |
| 4 | A | Siemens Biograph TruePoint | 336x336 | 2.04x2.04 | 2.50 | OSEM3D+PSF | 3i21s |
| 24 | A | Siemens Biograph TruePoint | 336x336 | 2.04x2.04 | 2.00 | OSEM3D+PSF | 3i21s |
| 1 | A | Siemens Biograph TruePoint | 336x336 | 2.04x2.04 | 4.00 | OSEM3D | 4i8s |
| 1 | A | Siemens Biograph TruePoint | 256x256 | 2.67x2.67 | 3.00 | OSEM2D | 4i8s |
| 1 | A | GE Discovery LS | 128x128 | 3.91x3.91 | NA | OSEM | NA |
| 1 | B | Siemens Biograph mCT | 400x400 | 2.04x2.04 | 2.50 | OSEM3D+PSF+TOF | 4i21s |
| 1 | B | Siemens Biograph mCT | 400x400 | 2.04x2.04 | 2.50 | OSEM3D+PSF | 4i21s |
| 1 | C | Siemens Biograph TruePoint | 336x336 | 2.04x2.04 | 2.00 | OSEM3D+PSF | 3i21s |
| 2 | C | Siemens Biograph TruePoint | 168x168 | 4.07x4.07 | 5.00 | OSEM2D | 4i8s |
| 1 | C | Siemens Biograph Vision | 440x440 | 1.65x1.65 | No filter | OSEM3D+PSF+TOF | 3i5s |
| 3 | C | Siemens Biograph mCT | 400x400 | 2.04x2.04 | 2.50 | OSEM3D+PSF+TOF | 2i21s |
| 1 | D | Siemens Biograph TruePoint | 336x336 | 2.04x2.04 | 3.00 | OSEM+PSF | 4i21s |
| 1 | E | Siemens Biograph mCT | 200x200 | 4.07x4.07 | 3.00 | OSEM3D+PSF+TOF | 2i21s |
| 1 | F | Siemens Biograph Vision | 440x440 | 1.65x1.65 | No filter | OSEM3D+PSF+TOF | 415s |
| 1 | G | GE Discovery MI | 256x256 | 2.73x2.73 | NA | Q.Clear | NA |
| 2 | H | Philips Gemini TF TOF | 144x144 | 4x4 | NA | BLOB-OS-TF | NA |
| 1 | I | Philips Gemini TF TOF | 144x144 | 4x4 | NA | BLOB-OS-TF | NA |
| 1 | J | GE Discovery 710 | 128x128 | 5.47x5.47 | NA | VPFX-S (OSEM3D+TOF) | NA |
| 1 | J | GE Discovery RX | 128x128 | 5.42x5.42 | NA | 3D IR | NA |
| 1 | J | GE Discovery 710 | 256x256 | 2.73x2.73 | NA | Q.Clear (β = 500) | NA |
| 1 | J | GE Discovery 690 | 256x256 | 2.73x2.73 | NA | VPFX-S (OSEM3D+TOF) | NA |
| 1 | J | GE Discovery MI | 256x256 | 2.73x2.73 | NA | Q.Clear (β = 500) | NA |

**Table S2. Data on reconstruction parameters from Copenhagen University Hospital-Rigshospitalet (Hospital A) and from other centers. Abbreviations: i = iterations, mm = millimeter, s = subsets.**
